# Supplementary figures and images for: cis-Decalin-containing tetramic acids as inhibitors of insect steroidogenic glutathione S-transferase Noppera-bo
Source: PLoS One. 2023 Aug 31;18(8):e0290851. doi: 10.1371/journal.pone.0290851 (PMC10470909; doi:10.1371/journal.pone.0290851)

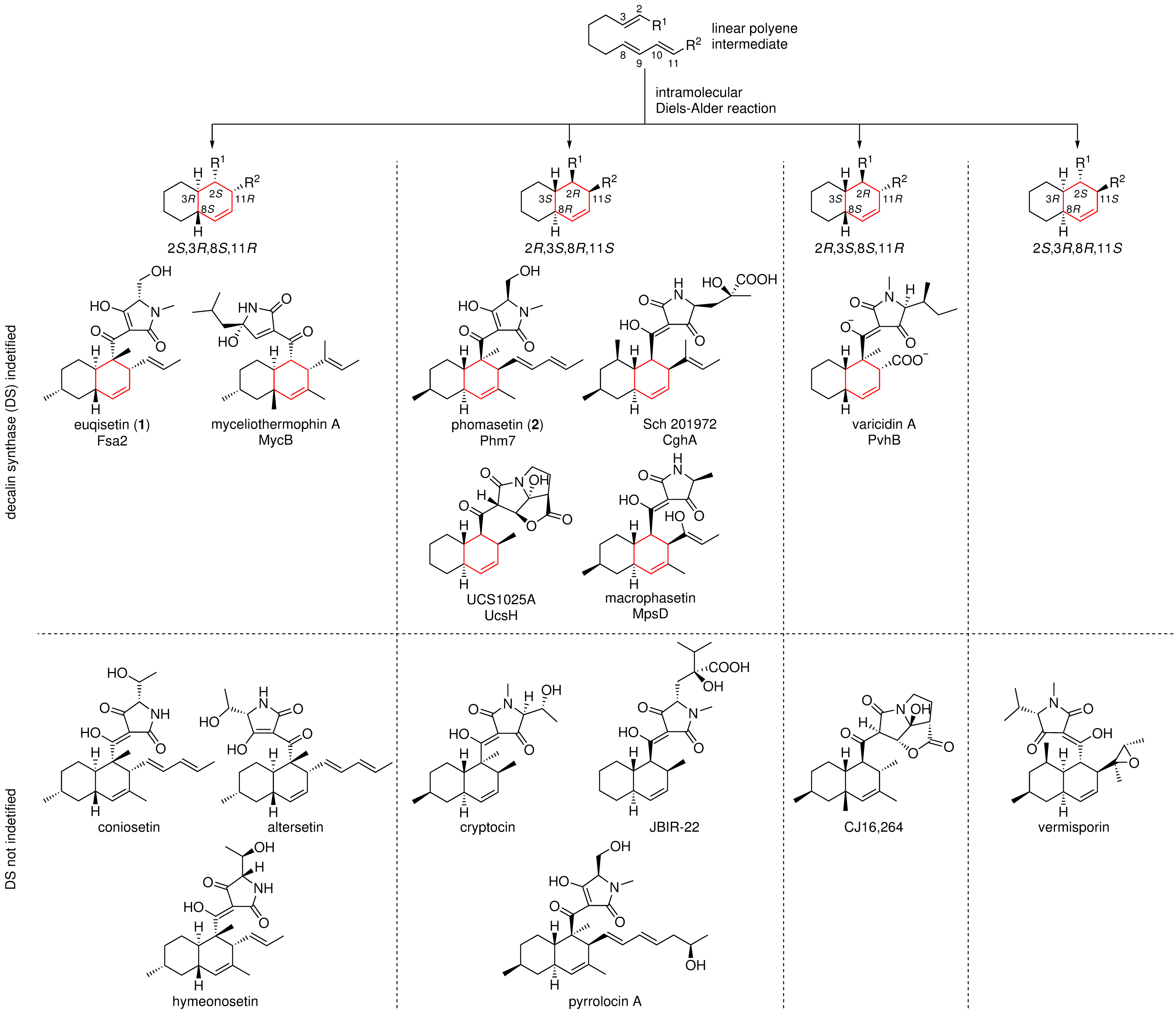

Supplement: S1 Fig — Decalin-containing tetramic acids were divided into four groups based on the configuration of decalin, which is formed from linear polyene intermediates via an intramolecular Diels–Alder reaction by decalin synthase (DS). Compounds and their corresponding DSs identified and those whose DS or biosynthetic gene cluster were not identified are shown in the top and bottom panels, respectively. (TIF) [file pone.0290851.s002.tif]

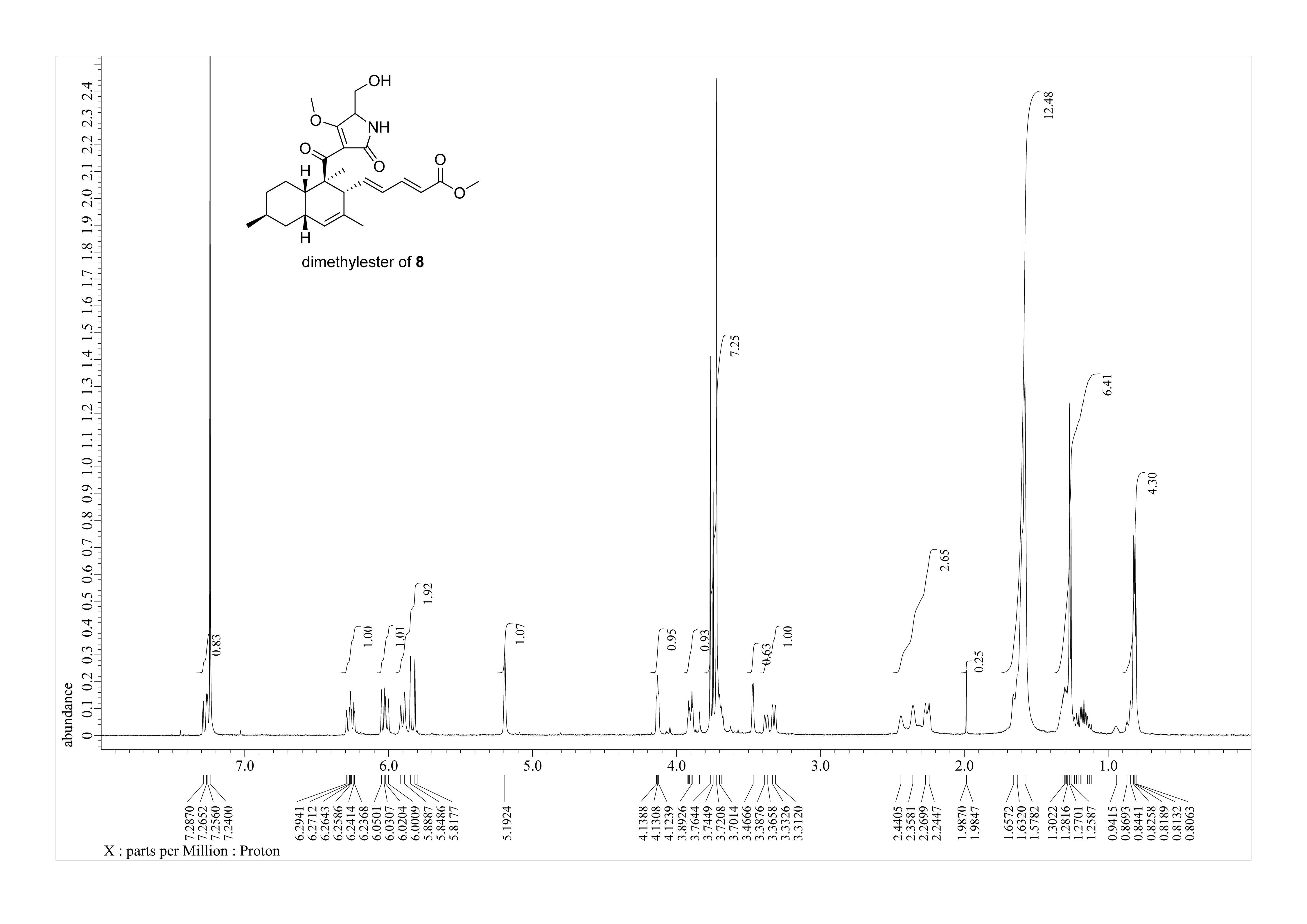

Supplement: S2 Fig — (TIF) [file pone.0290851.s003.tif]

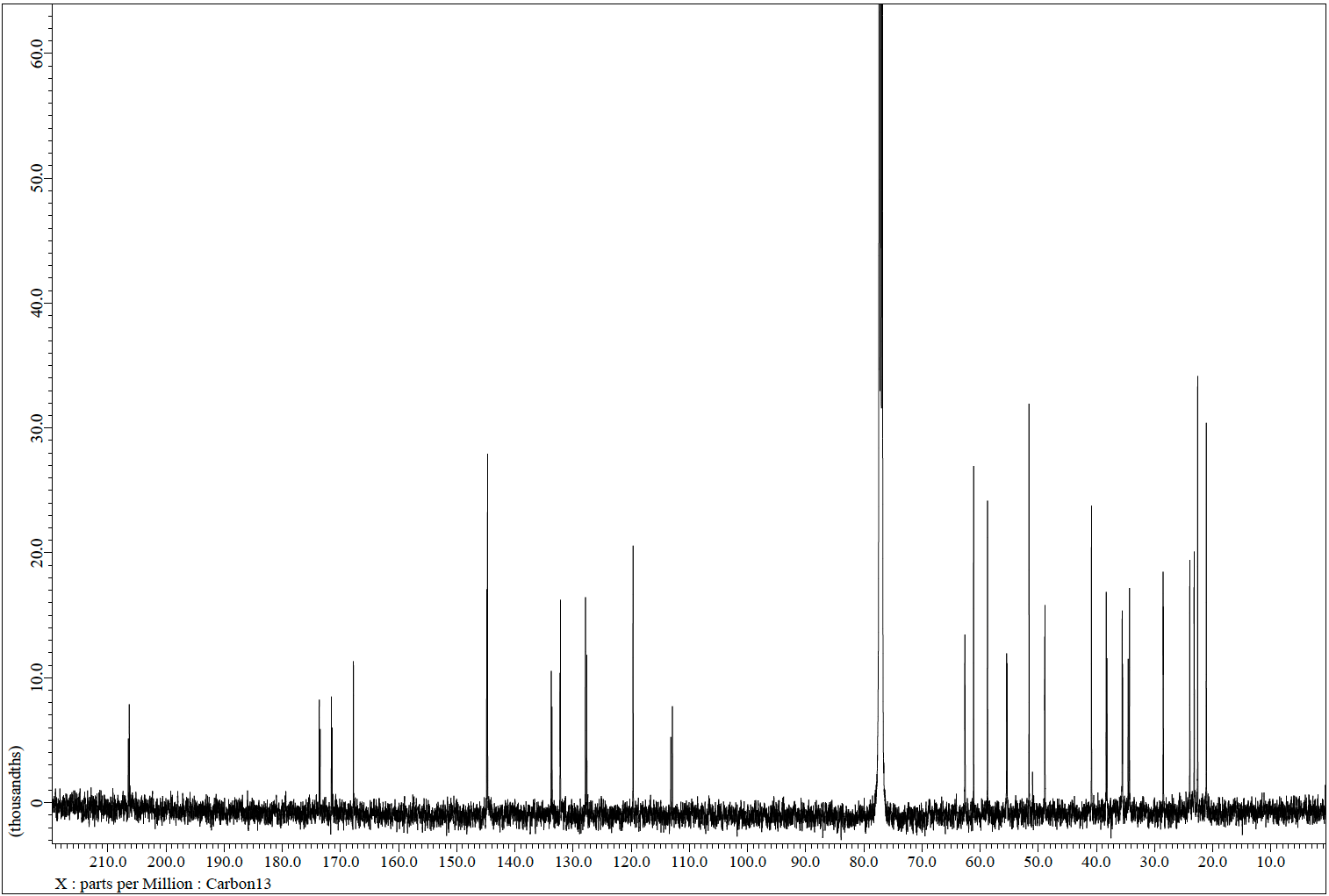

Supplement: S3 Fig — (TIF) [file pone.0290851.s004.tif]

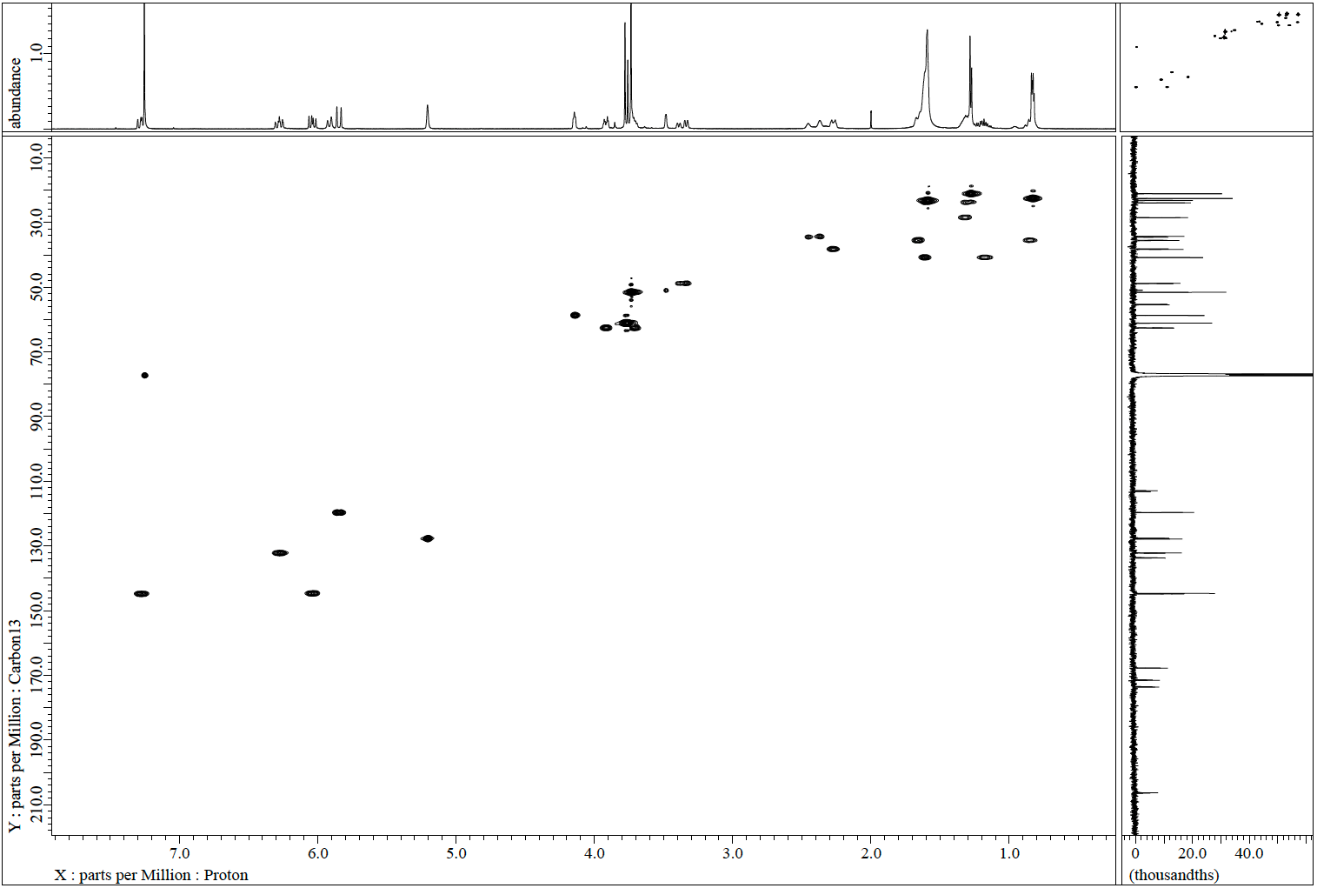

Supplement: S4 Fig — (TIF) [file pone.0290851.s005.tif]

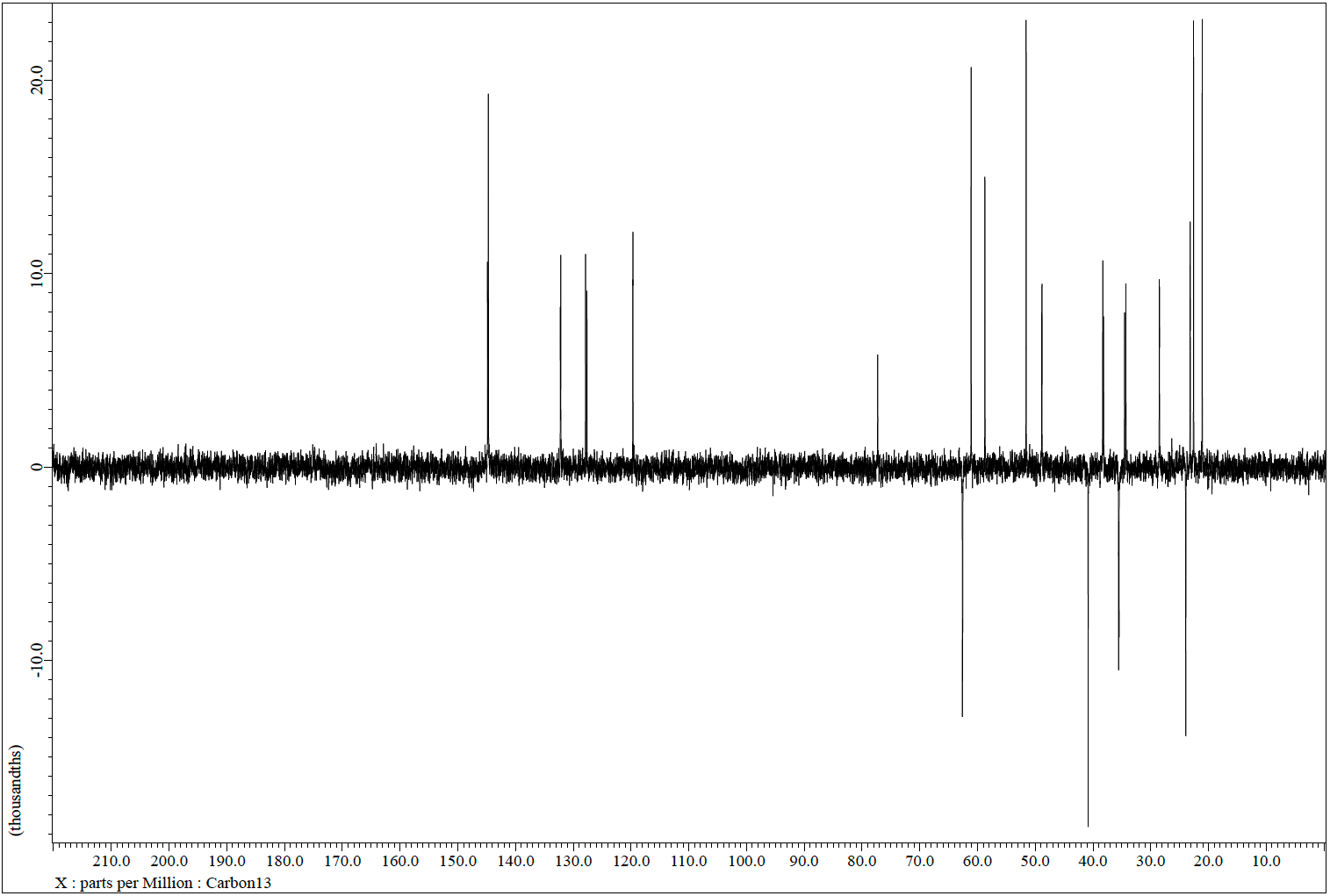

Supplement: S5 Fig — (TIF) [file pone.0290851.s006.tif]

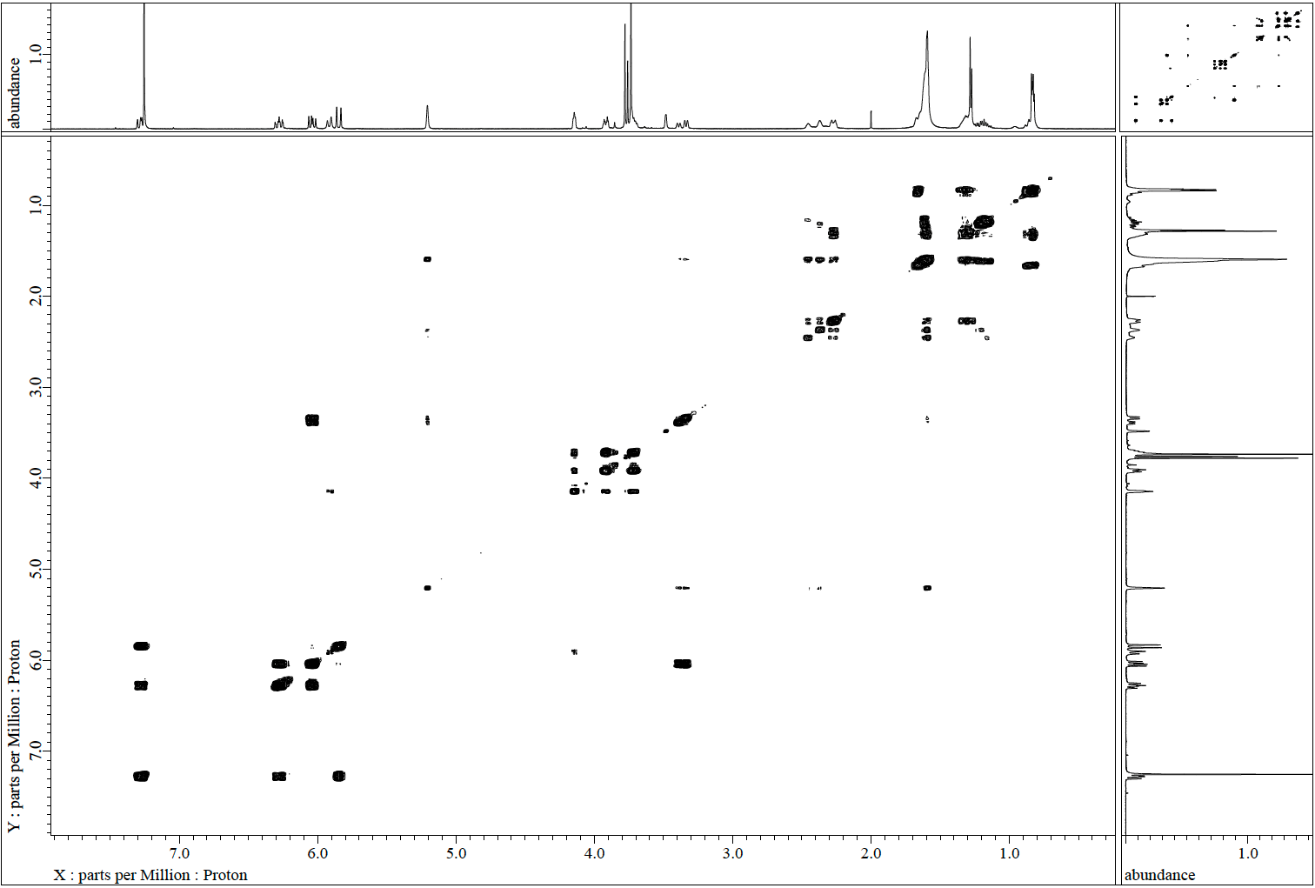

Supplement: S6 Fig — (TIF) [file pone.0290851.s007.tif]

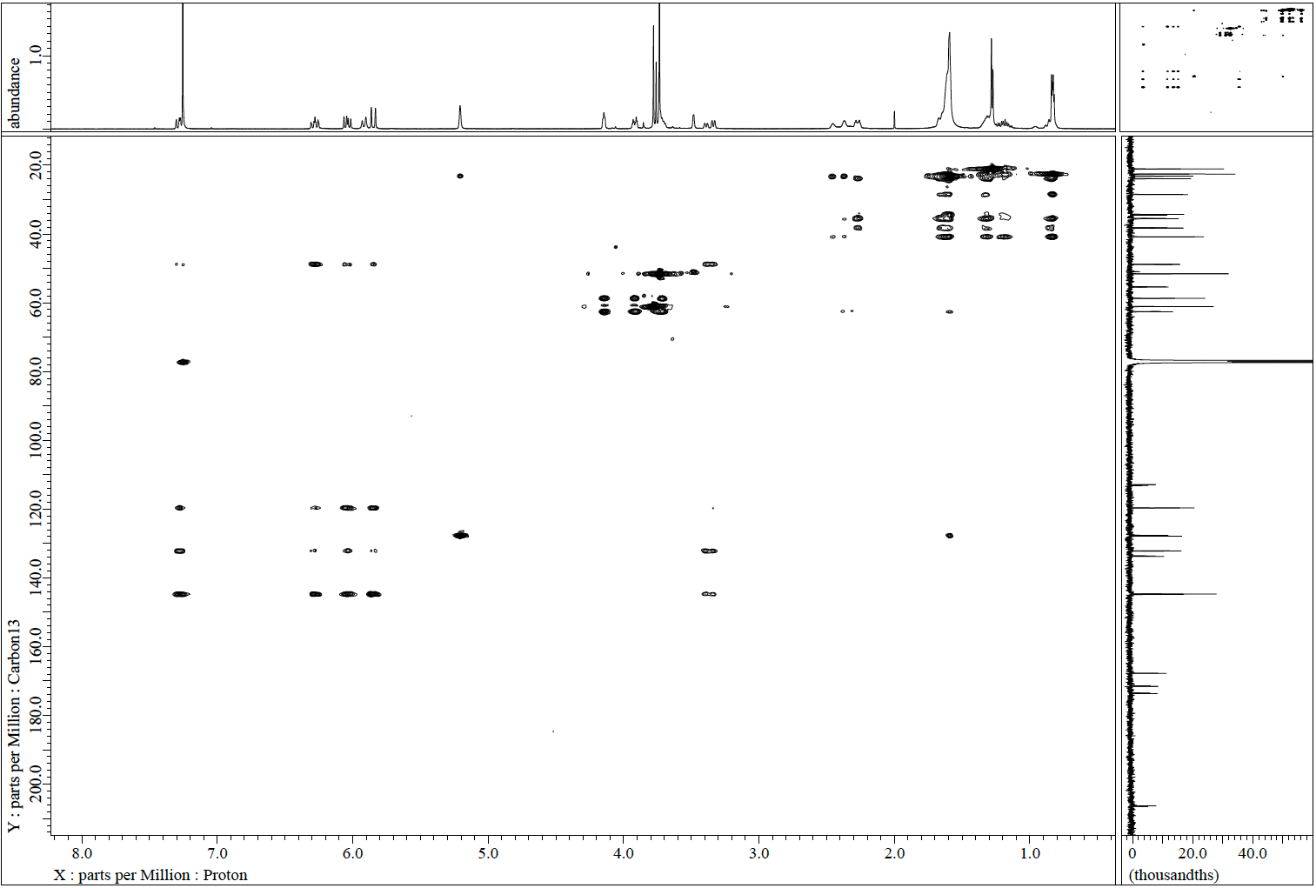

Supplement: S7 Fig — (TIF) [file pone.0290851.s008.tif]

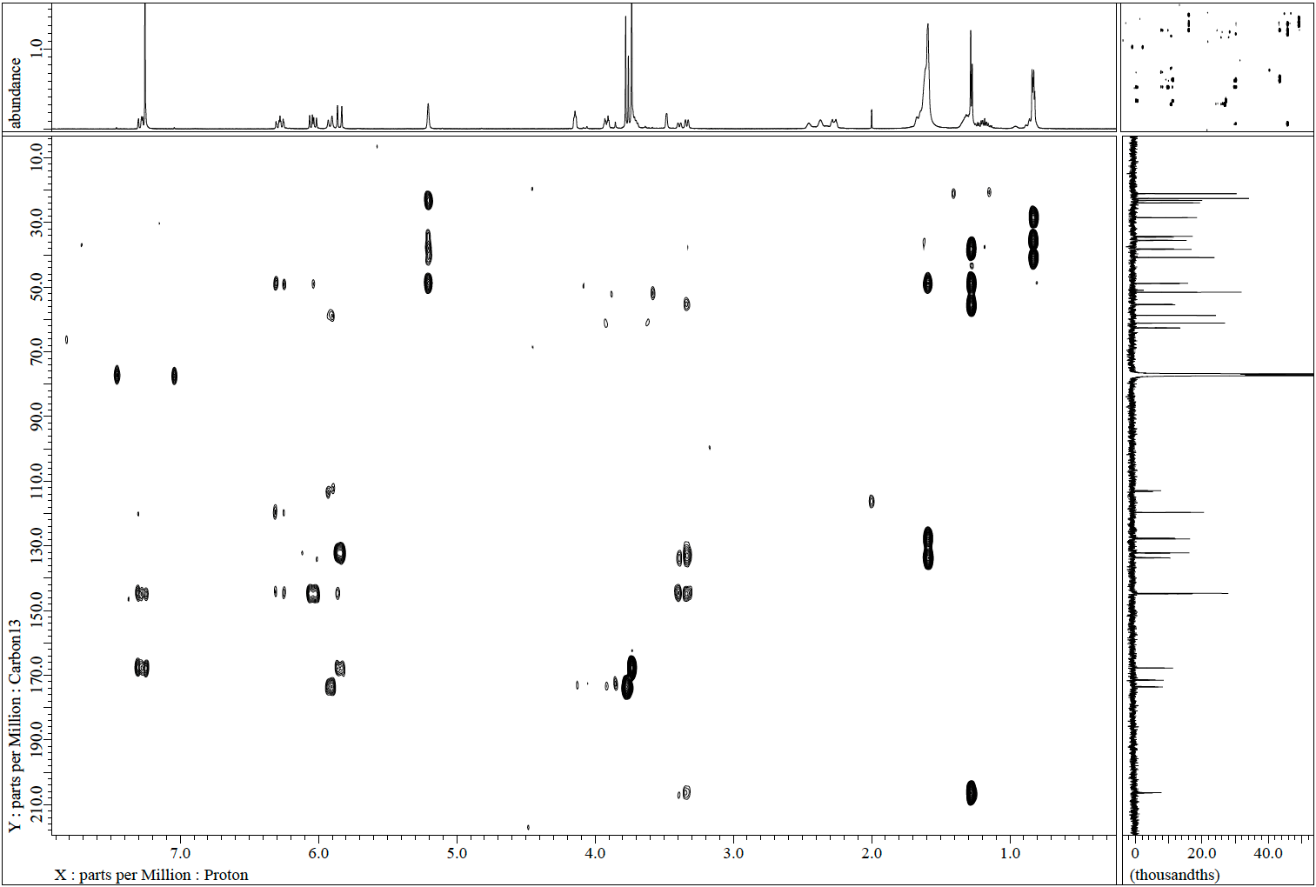

Supplement: S8 Fig — (TIF) [file pone.0290851.s009.tif]

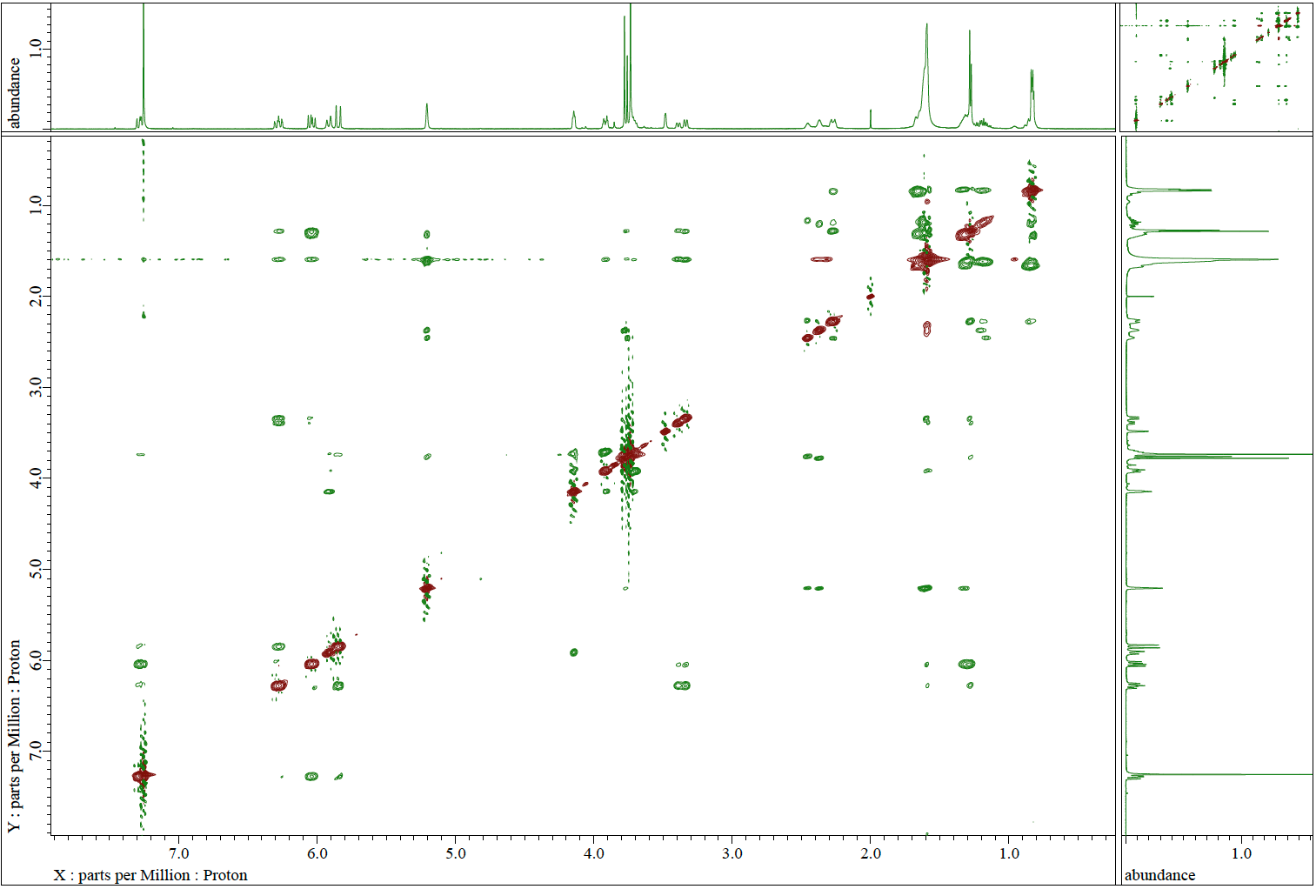

Supplement: S9 Fig — (TIF) [file pone.0290851.s010.tif]

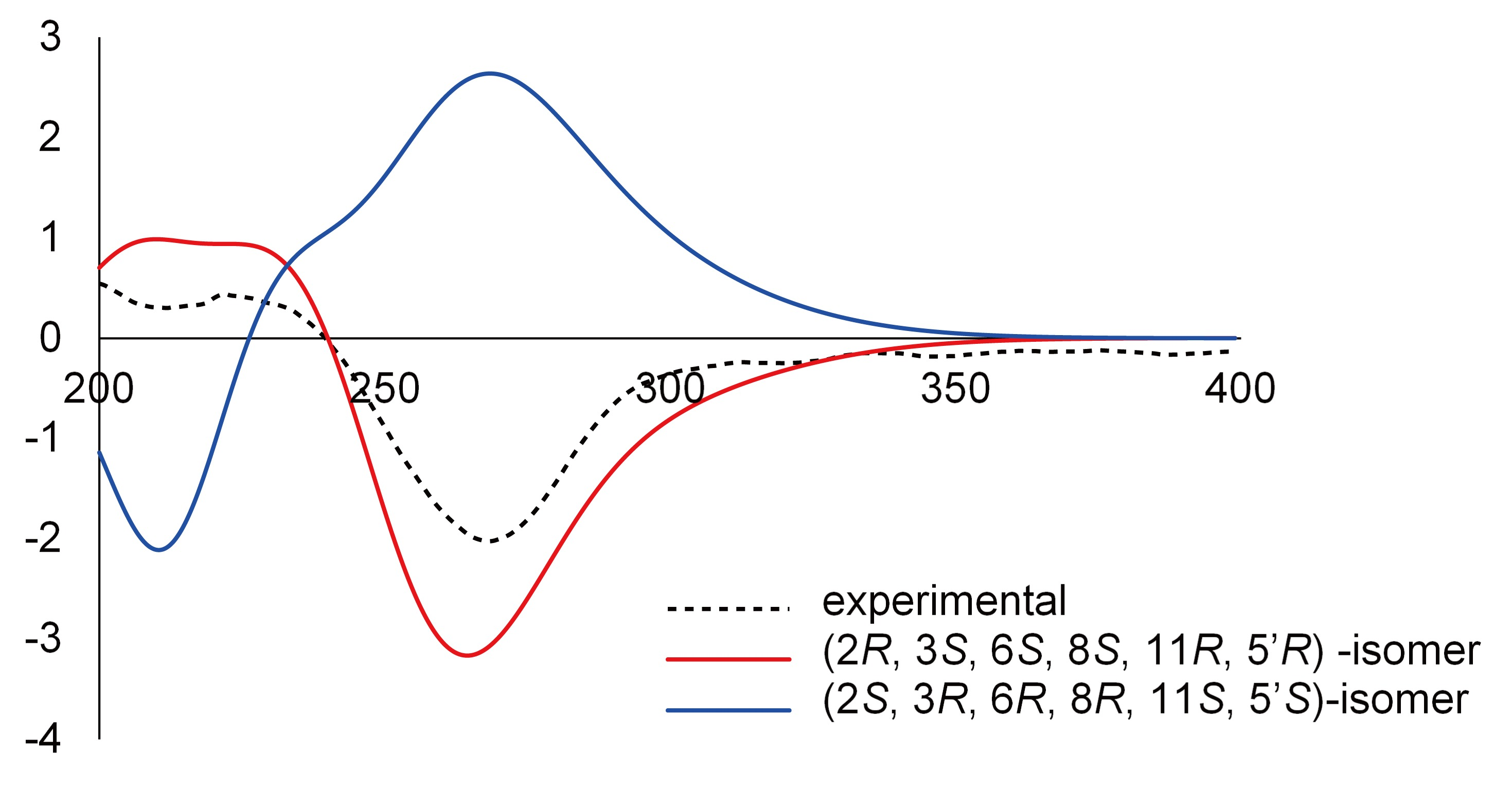

Supplement: S10 Fig — (TIF) [file pone.0290851.s011.tif]

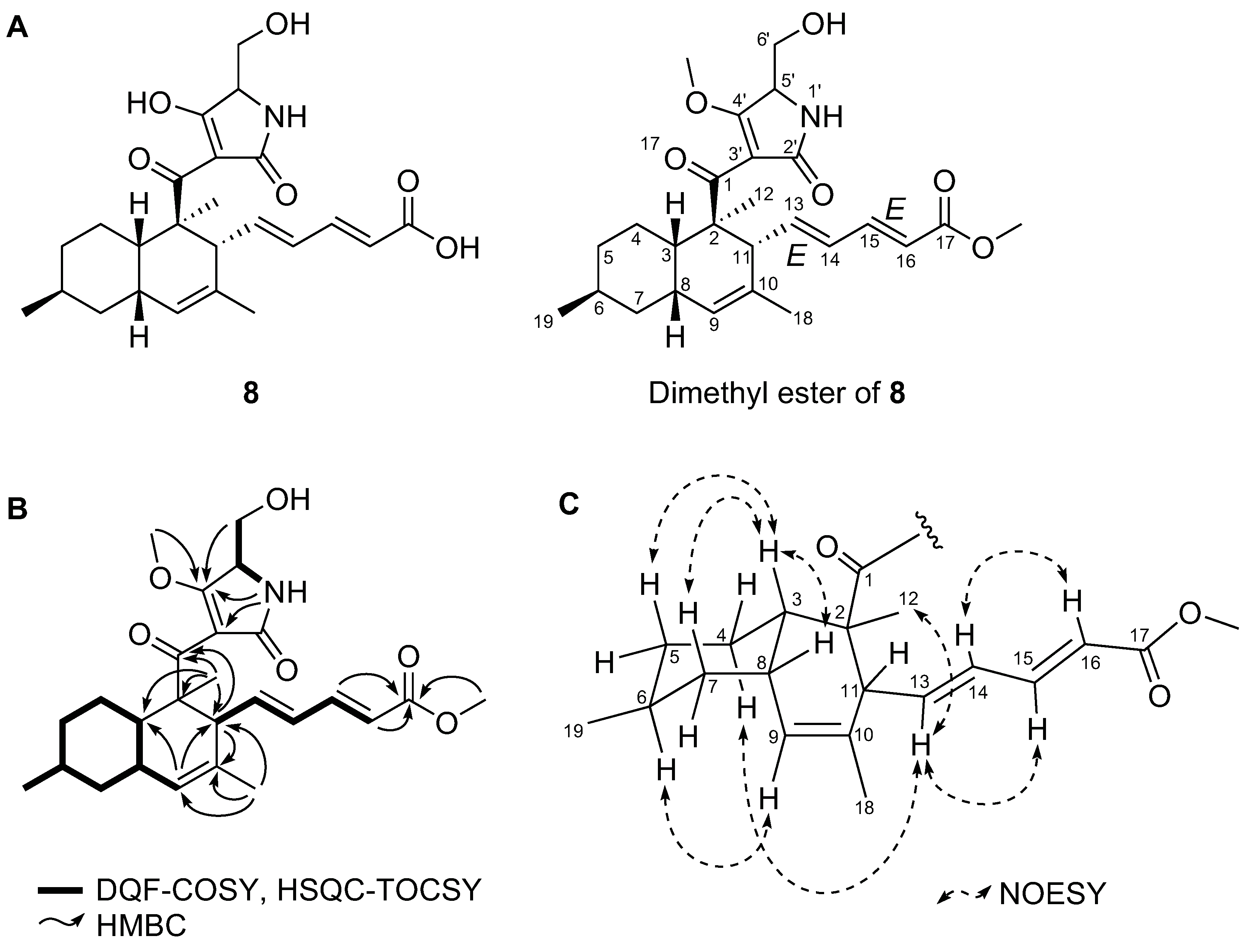

Supplement: S11 Fig — (TIF) [file pone.0290851.s012.tif]

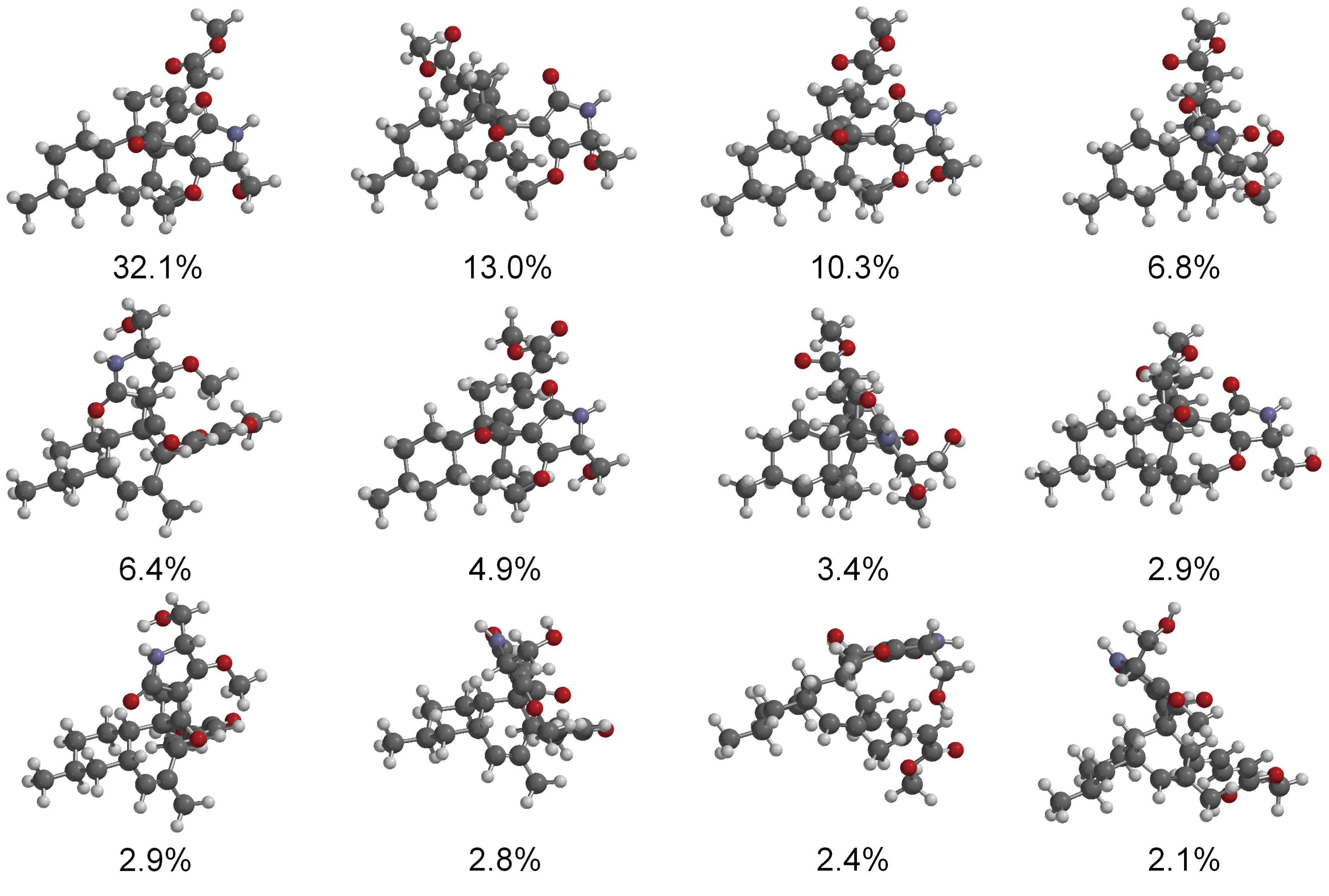

Supplement: S12 Fig — (TIF) [file pone.0290851.s013.tif]

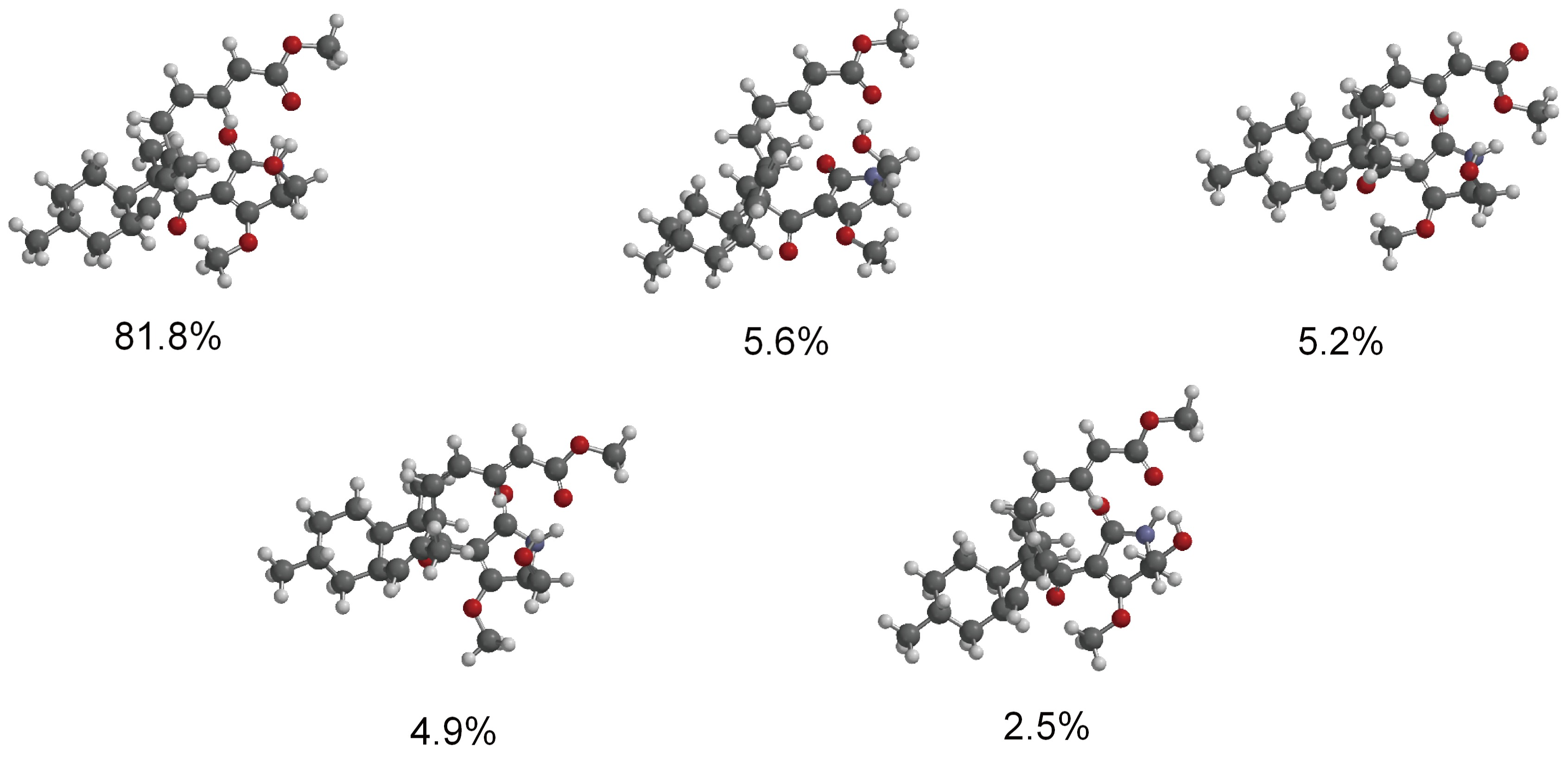

Supplement: S13 Fig — (TIF) [file pone.0290851.s014.tif]

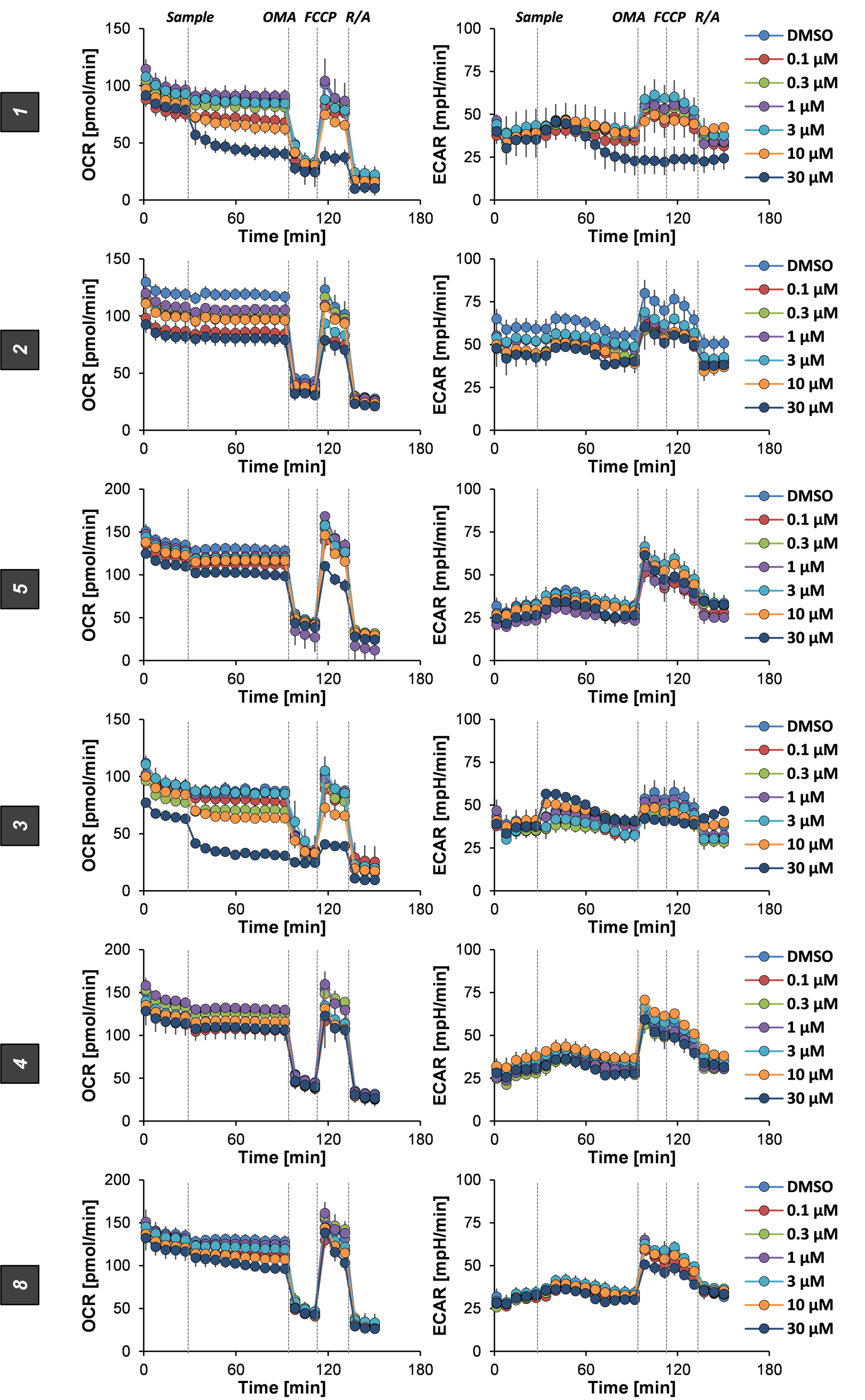

Supplement: S14 Fig — Real-time measurements of the OCR and ECAR in HeLa cells were performed after treating the cells with different concentrations of the test samples. To perform the Seahorse XF Cell Mito Stress Test, the cells were treated with oligomycin A (OMA, 1 μM), FCCP (0.125 μM), and rotenone/antimycin A (R/A, 1 μM each) at the indicated times. Data are mean ± s.d. (n = 3 technical replicates) from one representative experiment out of three independent experiments. (TIF) [file pone.0290851.s015.tif]

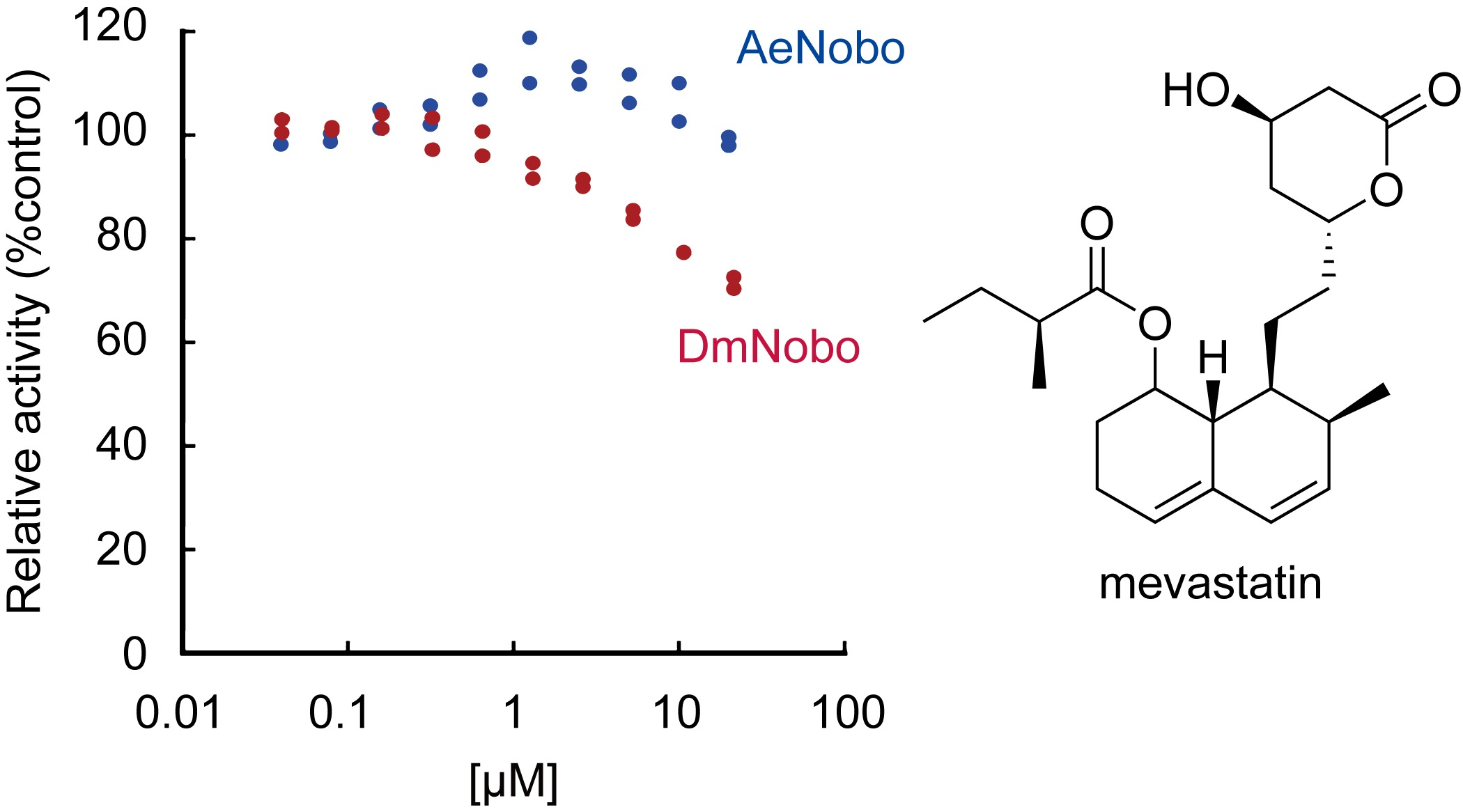

Supplement: S15 Fig — Inhibition of GSH conjugation activities of wild-type DmNobo (red dots) and AeNobo (blue dots) using 3,4-DNADCF was measured in the presence of mevastatin. Relative activity was defined as the ratio of activity between the respective proteins without the compounds. (TIF) [file pone.0290851.s016.tif]
